# Rv2031c of *Mycobacterium tuberculosis*: a master regulator of Rv2028–Rv2031 (HspX) operon

**DOI:** 10.3389/fmicb.2015.00351

**Published:** 2015-04-27

**Authors:** Khurram Mushtaq, Javaid A. Sheikh, Mohammed Amir, Nargis Khan, Balvinder Singh, Javed N. Agrewala

**Affiliations:** Institute of Microbial Technology, Council of Scientific and Industrial ResearchChandigarh, India

**Keywords:** protein modeling, molecular docking, phylogenetic analysis, protein interaction network analysis, latent TB infection

## Abstract

Genes belonging to the same operon are transcribed as a single mRNA molecule in all prokaryotes. The genes of the same operon are presumed to be involved in similar metabolic and physiological processes. Hence, computational analysis of constituent proteins could provide important clues to the functional relationships within the operonic genes. This tends to be more fruitful in the case of *Mycobacterium tuberculosis (Mtb),* considering the number of hypothetical genes with unknown functions and interacting partners. Dramatic advances in the past decade have increased our knowledge of the mechanisms that tubercle bacilli employ to survive within the host. But the phenomenon of *Mtb* latency continues to baﬄe all. Rv2031c belonging to dormancy regulon of *Mtb* is predominantly expressed during latency, with myriad immunological roles. Thus we attempted to analyze the operon comprising Rv2031c protein to gain insights into its role during latency. In the current study, we have carried out computational analysis of proteins encoded by genes known to be a part of this operon. Our study includes phylogenetic analysis, modeling of protein 3D structures, and protein interaction network analysis. We describe the mechanistic role in the establishment of latency and regulation of DevS–DevR component system. Additionally, we have identified the probable role of these proteins in carbohydrate metabolism, erythromycin tolerance, and nucleotide synthesis. Hence, these proteins can modulate the metabolism of *Mtb* inside the host cells and can be important for its survival in latency. The functional characterization and interactome of this important operon can give insight into its role during latency along with the exploitation of constituent proteins as drug targets and vaccine candidates.

## Introduction

*Mycobacterium tuberculsosis* (*Mtb)* is one of the most successful pathogen owing to its capability to persist within host in latent state for a longer period. Nearly one-third of human population worldwide is infected with the latent form of *Mtb*. Latest reports suggest that about 1.5 million people died from this dreadful disease in 2013 (WHO, 2014). *Mtb* efficaciously evades immune system and therefore persists for years in humans in a clinically latent state. Consequently, majority of active TB cases arise due to reactivation of latent TB. The latent TB has been linked to hypoxic conditions and to survive, *Mtb* must adapt itself to low oxygen environment. Therefore, understanding the transcriptional machinery of *Mtb*, operated during latency will be quite helpful in abolishing its survival. Predicting functions of proteins expressed by a hypoxia responsive operon through computational method can be a logistic or primary approach in identifying novel targets to disrupt pathogens machinery and thereby impairing its survival.

The *Mtb* Rv2031c α-crystallin gene expression is dramatically induced at reduced oxygen tensions. Its role is well established in the survival of mycobacteria in non-replicating phase (Cunningham and Spreadbury, 1998). The importance of Rv2031c, known as alpha-crystallin (HspX), is exemplified by the fact that depletion of this protein deteriorates the *Mtb* tolerance to anaerobiosis. Thus, it is invoked as a potential candidate facilitating survival of the pathogen during hypoxia, a situation that is similar to latency. Further, its role in impairing the immune response by blocking the differentiation of monocytes to DCs has been recently demonstrated (Siddiqui et al., 2014). The operon is a specific functional organization of genes found in bacterial genomes. Understanding the characteristics of operon can provide the basis of understanding transcriptional regulation and the entire regulatory network of an organism. The *Mtb* genes Rv2028c, Rv2029c, Rv2030c, and Rv2031c, constitute a single operon named as Rv2028–Rv2031. Most of the genes within operons have interrelated functions. Thus, computational analysis of the genes belonging to Rv2028–Rv2031 operon may provide a new insight into mechanism crucial for the survival of *Mtb* inside the host.

In the current study, we have chosen Rv2031c, which is not only highly upregulated in hypoxic state, but also responsible for the universal cellular stress response (Yuan et al., 1996; Sherman et al., 2001). We have performed a detailed analysis of proteins encoded by Rv2028–Rv2031 operon using a combination of bioinformatics tools. Further, to support our results, we used information available in the literature (Sherman et al., 2001). Here, we report possible 3D structures and functional analysis of all four proteins Rv2028c, Rv2029c, Rv2030c, and Rv2031c generated using threading based approach. From the network analysis based on the protein–protein interaction, Rv2031c has emerged out to be a master regulator under hypoxic stress. Surprisingly, we found interaction with various *Mtb* proteins of unknown function, which will add new dimension to our understanding of pathways operated in latent phase of TB.

## Materials and Methods

### Data Acquisition

Protein sequences belonging to Rv2028c–Rv2031c operon of *Mtb* were downloaded in FASTA format from UniProtKB^1^ in December, 2013 (UniProt, 2008) and were then subjected to various *in silico* tools described below.

### Phylogenetic Analysis and Orthology Search

In order to gain insights into evolutionary pattern of this operon among selected mycobacterial species (Supplementary Table S1), we adopted a phylogenetics based method. These species were selected on the basis of their different level of pathogenicity and host restriction. Protein sequences for each species were retrieved using BlastP of NCBI^2^ (Altschul et al., 1990) and cladograms were constructed using Clustal omega^3^ (Sievers et al., 2011) with default parameters. The hits showing percent identity above 35% were considered as significant, for orthology search sequence identity between 20 and 35% was considered as probable orthology and below 20% as non-orthology. (Rost, 1999). Bootstrap analysis to evaluate reliability of phylogenetic trees was performed for 1000 iterations using MEGA 6.0 (Tamura et al., 2013).

### Gene Ontology Term (GO) Enrichment

Complete GO annotation for each protein was obtained using UniProt–GOA^4^ (Barrell et al., 2009). These GO terms were then summarized and clustered into subsets using REVIGO^5^ (Supek et al., 2011) to remove very general GO terms like “functional group transfer.”

### Domain Search

To identify presence of functional domains the protein sequences were queried against CDD^6^ (Marchler-Bauer et al., 2013), ProDom^7^ (Bru et al., 2005), and InterPro^8^ (Mitchell et al., 2014).

### Prediction of Sub-Cellular Localization

In order to gain insight into where a particular protein is sorted after synthesis, we have used protein sub-cellular prediction method, WoLF PSORT^9^ (Nakai and Horton, 1999) that makes predictions based on known strong sorting signal motifs and amino acid content.

### Prediction of Transmembrane Helices

To identify the presence of transmembrane regions, protein sequences were submitted to TMHMM^10^ (Krogh et al., 2001) which makes predictions based on Hidden Markov model and TMpred^11^ (Hofmann and Stoffel, 1993) based on statistical analysis algorithm.

### Prediction of Signal Peptides

In order to identify proteins that can be a part of mycobacterial secretion system, we performed N-terminal signal peptide search using SignalP^12^ (Petersen et al., 2011) which is based on artificial neural networks.

### 3D Structure Modeling

The protein 3D structures were predicted using I-TASSER^13^ which uses threading approach for structural modeling (Zhang, 2008). Only the modeled structures having highest confidence score were considered significant. The modeling web-server also collaborates closely with other software like COFACTOR (predicts EC number; Roy et al., 2012) which gives information about the model. These results were taken into consideration for further study.

### Molecular Docking

Sequence analysis revealed the presence of ATP-binding sites in RV2028c and Rv2029c. To confirm the ATP-dependent activity of these proteins molecular docking was performed using AutoDock Vina (Trott and Olson, 2010) and results were analyzed using Autodock tools and Ligplot+ (Laskowski and Swindells, 2011).

### Protein Interaction Network Analysis

Probable protein–protein interaction data was retrieved using string database^14^ (Jensen et al., 2009). Only the functional partners showing high confidence (score 0.7 or more) were considered as significant. Protein interaction network analysis was performed using cytoscape (Shannon et al., 2003).

### Antigenicity Index

The antigen index of proteins was calculated using VaxiJen 2.0^15^ (Doytchinova and Flower, 2007) at the default threshold value of 0.4. It is based on an alignment independent approach and predicts probable antigens solely based on physiochemical properties of protein sequence.

## Results

### Phylogenetic Analysis

**FIGURE 1:**
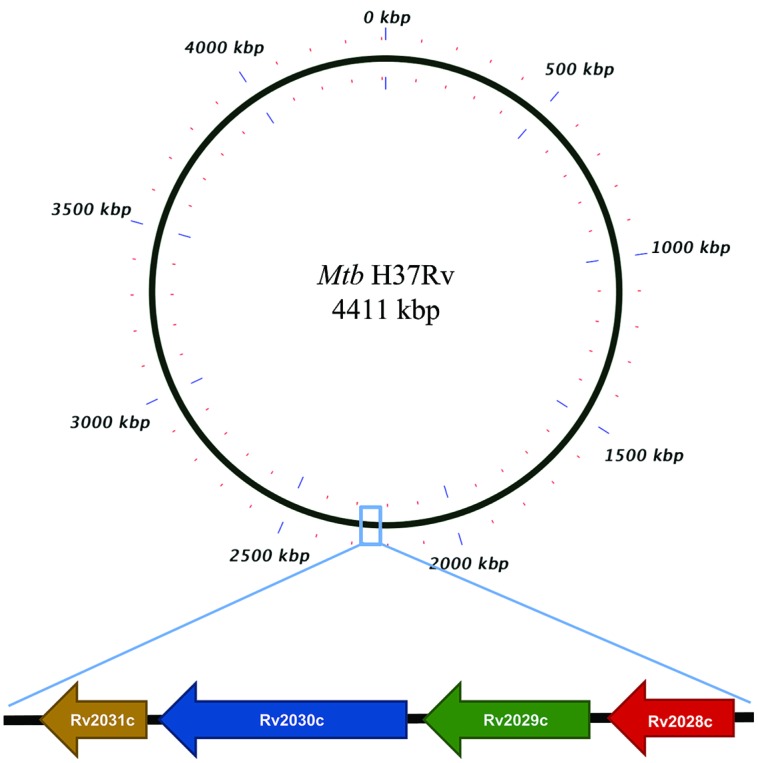
**Pictorial respresentation of Rv2028c–Rv2031c operon.** Diagram represents genomic organization of R2028c–Rv2031c operon with constituent genes and their names represented by arrows (not to scale).

**FIGURE 2:**
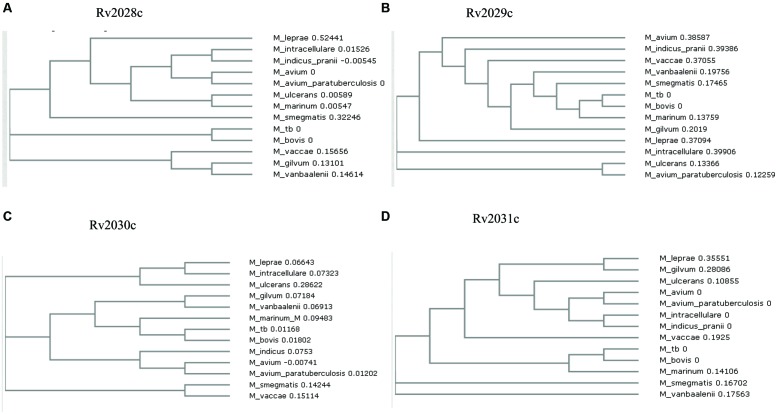
**Phylogenetic profile of proteins encoded by Rv2028c–Rv2031c operon.** Phylogenetic trees for **(A)** Rv2028c; **(B)** Rv2029c; **(C)** Rv2030c; **(D)** Rv2031c constructed using neighbor joining method. *Mycobacterial* species sharing common ancestry are placed in the sister groups forming different taxon.

The conservation in protein sequences can reflect their probable functional role in an organism. Therefore, to identify the evolutionary conserved pattern of selected operon (**Figure 1**), a phylogenetic profile based approach was used. Phylogenetic tree constructed using the neighbor joining method placed *Mtb* and *M. bovis* as sister group in case of all proteins; *M. leprae* and* M. intracellulare* were, however, placed as outgroups to *Mtb* (**Figures 2A–D**). Moreover, It was observed that all four proteins were identical in *Mtb* and *M. bovis* (percent sequence identity 97.03–100%; **Figures 2A–D**, Supplementary Figures S1A–D). In contrast, *M. leprae* and *M. intercellulare* showed no clear orthology to *Mtb* proteins (Supplementary Figures S1A–D). These results signify that *Mtb* and *M. bovis* show co-evolution and are more closely related with respect to this operon. However, *M. leprae* and *M. intracellulare* are distantly related during evolution. Additionally, *M. smegmatis* and *M. givlum* had orthologs for all four proteins (Supplementary Figures S1A–D). Rv2028c (Supplementary Figure S1A) and Rv2030c (Supplementary Figure S1D) orthologs were detected in *M. ulceranus* and *M. indicuspranii*, respectively, (Supplementary Figures S1A–D). Further consensus phylogenetics trees were plotted after 1000 bootstrap iterations and same trend was observed, substantiating the reliability of our results (Supplementary Figures S2A–D).

### Domain Search

Identification of known functional domains is useful in the characterization of proteins with unknown function. In this study, Rv2029c was found to have a phosphofructokinase like domain (Supplementary Figure S3B), suggesting its probable role in fructose utilization by *Mtb*. In addition, it was found to have putative substrate and ATP binding sites as well with an *e*-value of 4.50e-103, which can be critical for its functionality as an enzyme. On the other hand Rv2030c was found to have phosphoribosyltransferase (PRT) domain (Supplementary Figure S3C) and the main product of PRTase catalyzed reactions are key metabolite connecting nucleotide synthesis and salvage pathway. The database search also revealed that Rv2030c belonged to erythromycin esterase superfamily, the members of which are critical for conferring erythromycin tolerance. These results indicate that RV2029c is an important protein for carbohydrate metabolism while Rv2030c for nucleotide metabolism and erythromycin tolerance.

### Prediction of Transmembrane Helices

Ambiguity in subcellular localization of HspX between cell wall and plasma membrane (Gu et al., 2003; Mawuenyega et al., 2005) prompted us to investigate whether these proteins have transmembrane region. As it can help us to identify proteins forming mycomembrane translocon and may further assist us to gain insights into probable functionality of these proteins. However, none of the protein used in this study was found to form transmembrane helix. These results rule out the possibility of involvement of these proteins in secretory mechanism as well (**Table 1**).

**Table 1:** Functional annotation of proteins expressed by genes under Rv2028c–Rv2031c operon.

Gene	Protein name	Known function (from literature)	Probable function (from this study)	Function derived from	Subcellular localization	Transme-mbrane helices and signal peptides	Antigenicity
*Rv2028c*	Universal stress protein	Induced in response to hypoxia, low levels of nitric oxide (NO), and CO	Response to stress, probably involved in activation of DevR–DevS regulatory system	Gene ontology, domain analysis, protein–protein interaction, literature	Plasma membrane	None	Probable Antigen
*Rv2029c*	Putative phosphofructokinase	Induced in response to hypoxia, low levels of NO, and CO	Probably involved in carbohydrate metabolic process and phosphorylation	Gene ontology, domain analysis, protein–protein interaction, literature	Plasma membrane	None	Probable antigen
*Rv2030c*	Uncharacterized protein	Induced in response to hypoxia, low levels of NO, and CO	Probably involved in nucleoside metabolic process and response to antibiotic	Gene ontology, domain analysis, literature	Cytosol	None	Non-antigen
*Rv2031c*	Alpha-crystallin	Acts as a chaperone	Protein folding, protein binding, response to nitrostative stress, response to starvation, growth inside host organelle	Gene ontology, domain analysis, protein–protein interaction, literature	Cell wall	None	Probable antigen

Probable functional prediction of Rv2028c–Rv2031c operon analyzed employing different programs. Diverse probable roles apart from the known functions are summarized along with the methods used.

### 3D Structural Analysis

**FIGURE 3:**
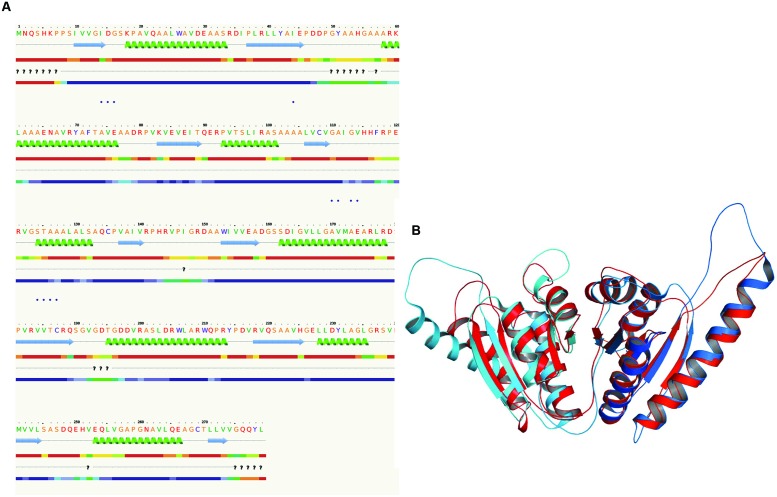
**Presence of Rossmann like α/β fold in Rv2028c.** Secondary and tertiary structure analysis reveals the presence of Rossmann like α/β fold, which is found in proteins with stress enduring activity. **(A)** Figure represents alternate α and β secondary structures in Rv2028c; **(B)** superimposed tertiary structures of Rv2028c (red) and Rv2623 of *Mycobacterium tuberculosis (Mtb*; blue) displayed in the cartoon.

**FIGURE 4:**
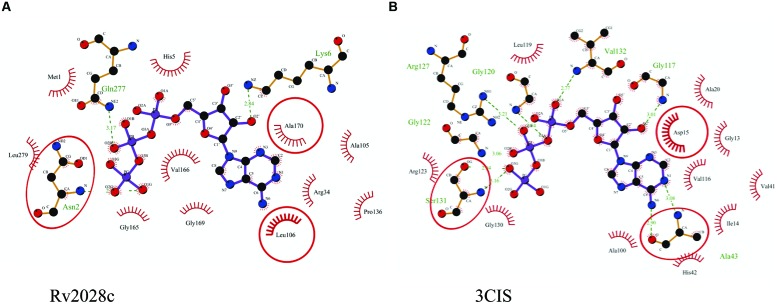
**Binding conformation of ATP docked to Rv2028c.** Figure shows interaction map of **(A)** docked ATP–Rv2028c complex; **(B)** crystal structure of ATP bound Rv2623 (PDBID: 3CIS) generated using LIGPLOT. Amino acids forming hydrogen bonds are shown in ball and stick representation and those forming close contacts are shown as arcs with spokes radiating toward ligand.

The 3D structure of proteins provide insights into their probable functions (Martin et al., 1998). The modeled structure of Rv2028c revealed a Rossmann like α/β fold (**Figures 3A,B**), which is found to be conserved in proteins with stress enduring activity of widely different in phylogenetic origins (Sousa and McKay, 2001; Kerk et al., 2003). The structural analysis revealed ATP binding sites similar to Rv2623 protein (PDBID: 3CIS) of *Mtb,* which is essential in regulating growth during latency in ATP-dependent manner (Drumm et al., 2009). To confirm whether Rv2028c follows an ATP-dependent mechanism or not, molecular docking was performed. Results indicated that Rv2028c forms a stable complex with ATP molecule in terms of non-covalent interactions and a free energy of -7.4 kcal/mol (**Figure 4A**). The docking conformation was compared with crystal structure of Rv2623 from *Mtb* that shares structural similarity to our model and has an ATP-dependent activity. It was observed that ATP binds to both proteins in a similar conformation and amino acids forming the active site also have comparable contacts with the ligand (**Figure 4B**). These results suggest the ATP-dependent activity of RV2028c.

**FIGURE 5:**
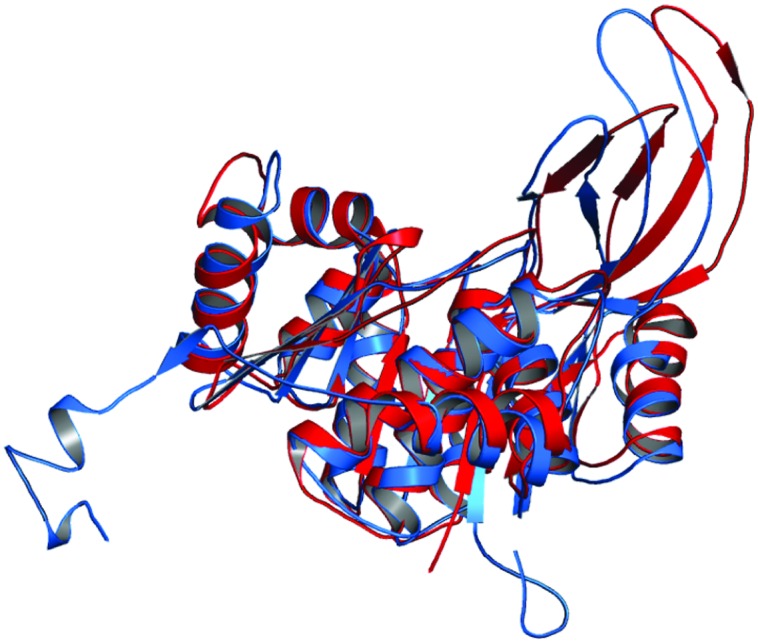
**Structural similarity between 6-phosphofructokinase isozyme 2 of *Escherichia coli* and Rv2029c of *Mtb*.** Figure shows superimposed crystal structure of 6-phosphofructokinase isozyme 2 from* E. coli* (blue) and modeled Rv2029c structure (red).

**FIGURE 6:**
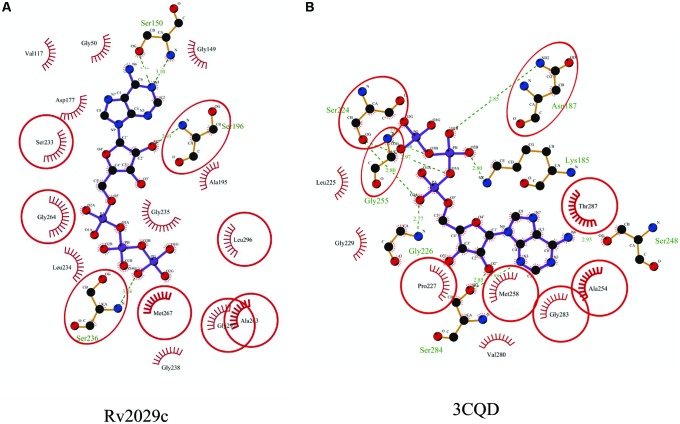
**Binding conformation of ATP docked to Rv2029c of *Mtb*.** Figure shows interaction map of **(A)** docked ATP–Rv2029c complex; **(B)** crystal structure of ATP bound 6-phosphofructokinase isozyme 2 of *E. coli* (PDBID: 3CQD) generated using LIGPLOT+. Amino acids forming hydrogen bonds are shown in ball and stick representation and those forming close contacts depicted as arcs with spokes radiating toward ligand.

Rv2029c shares structural similarity to 6-phosphofructokinase isozyme 2 of *Escherichia coli* (PDBID: 3CQD; **Figure 5**). Binding site prediction results indicated presence of ATP-binding sites in the protein. These results further suggest function of Rv2029c as fructokinase, which catalyzes ATP dependent conversion of fructose to fructose-6-phosphate. Rv2029c has a probable function as fructokinase, which catalyzes ATP dependent conversion of fructose to fructose-6-phosphate. To confirm the ATP-dependent activity, we utilized molecular docking approach. Top docking conformation with a free energy of -8.1 kcal/mol was then compared with ATP-bound crystal structure of 6-phosphofructokinase isozyme 2 of *E. coli* (PDBID: 3CQD; **Figure 6**). These results suggest the ATP-dependent activity of Rv2029c which is crucial for its role as a fructokinase.

**FIGURE 7:**
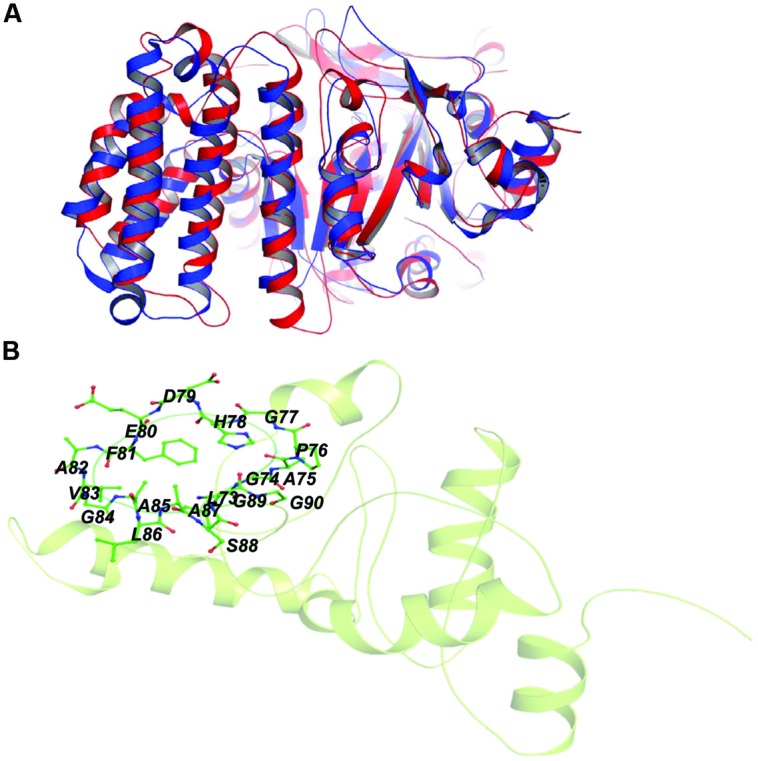
**Structural similarity between succinoglycan biosynthesis protein of *Bacillus cereus* and Rv2030c. (A)** Figure shows superimposed crystal structure of succinoglycan biosynthesis protein of *B. cereus* (blue) and modeled Rv2030c structure (red). **(B)** The putative PRPP motif which features two adjacent acidic residues surrounded by one or more hydrophobic amino acids is shown in stick representation.

Rv2030c showed a high structural similarity to succinoglycan biosynthesis protein of *Bacillus cereus* (RMS deviation -0.87 Å; PDBID: 3B55) that contains a PRTase domain and belongs to erythromycin esterase superfamily (**Figures 7A,B**). The data are in accordance with domain analysis results and further suggest the probable role of Rv2030c in nucleotide metabolism and erythromycin tolerance during latency.

**FIGURE 8:**
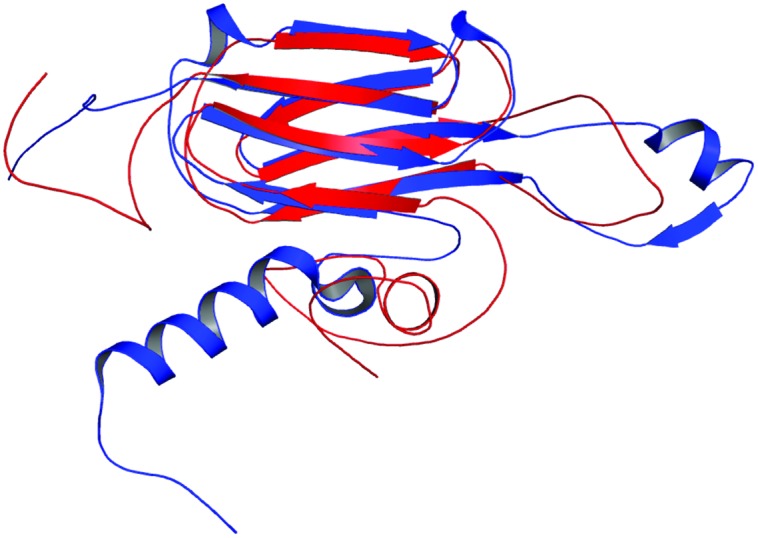
**Structural similarity between heat shock protein 16.0 of *Schizosaccharomyces pombe* and Rv2031c of *Mtb*.** Figure shows superimposed crystal structure of heat shock protein 16.0 of *S. pombe* (blue) and modeled Rv2031c structure (red).

In our study, the modeled structure of Rv2031c bore high structural similarity to heat shock protein 16.0 of *Schizosaccharomyces pombe* (RMS deviation - 2.00 Å; PDBID: 3W1Z), which has chaperonic activity that might play an instrumental role in maintaining proper folded structure of proteins at time of stress, thus promoting its survival (**Figure 8**). These results are in accordance with its already established role in protein folding and oligomerization (Chang et al., 1996; Yuan et al., 1996; Kennaway et al., 2005).

### Protein Interaction Network Analysis

**FIGURE 9:**
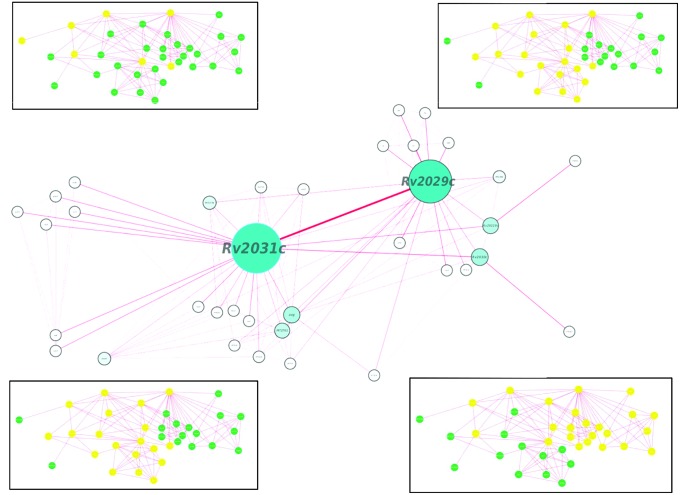
**Protein interaction network analysis of Rv2028c–Rv2031c proteins.** figure shows a protein interaction network of selected proteins. The node size and edge size are proportional to betweeness centrality and edge betweenness values, respectively. Immediate neighbors of individual nodes in the inset are highlighted in yellow in the order RV2028c, Rv209c, Rv2030c, and Rv2031c (from left to right).

Proteins usually work in synergy with each other *via* protein–protein interactions. Various cellular events like signal transduction, cell metabolism, transport across membranes, etc., take place through cumulative functioning of many interacting partners. In this study, proteins of our interest revealed direct or indirect role in response to stress, protein folding, expression of latency associated genes, carbohydrate metabolism, and phosphorylation. Altogether, Rv2031c emerged out as master regulator during hypoxic conditions as it exhibhitited maximum associating partners (based on betweenness centrality values; **Figure 9**). Besides, the interaction between Rv2031 and Rv2029c appeared to be quite crucial as both the proteins had maximum common interacting partners (based on edge betweenness values; **Figure 9**). These results indicate that the interaction between these two proteins is important in cumulatively regulating the whole network.

**FIGURE 10:**
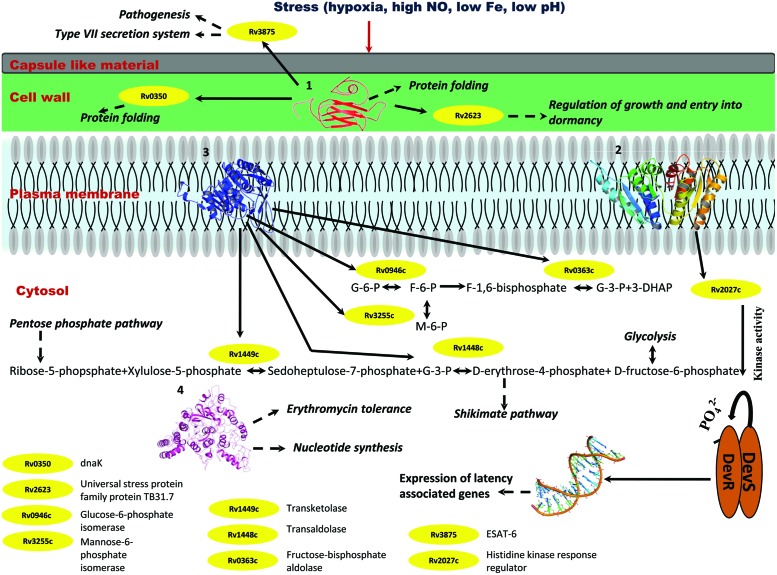
**Probable regulatory pathways encoded by Rv2028c–Rv2031c operon under stress conditions.** Figure represents different metabolic pathways and sub-cellular processes regulated by proteins encoded by Rv2028c–Rv2031c operon. The proteins under this operon are represented in a form of cartoon, whereas dotted lines and solid lines represent sub-cellular processes and protein–protein interactions, respectively. Different sub-cellular processes summarized in this figure are: (1) regulation of growth and protein folding by Rv208c (HspX); (2) regulation of DevS–DevR component system and subsequent expression of latency associated genes by Rv2029c (USP); (3) regulation of carbohydrate metabolism by Rv2030c (pfkb); (4) role of Rv2031c (conserved protein) in erythromycin tolerance and nucleotide synthesis.

Individually, Rv2028c was found to be associated with Rv2027c (histidine kinase response regulator/DosT), which has a kinase activity and plays an important role on signal transduction (**Figure 10**). The latter is responsible for activation of Rv3132c–Rv3133c (DevS–DevR) two component system and subsequent expression of latency associated genes (Sivaramakrishnan and de Montellano, 2013). Further, it was found to be uniquely associated with Rv2629 which is a hypothetical protein of dormancy regulon (**Figure 9**).

Rv2029c interacts with various enzymes that are directly involved in glycolysis like Rv0946c (glucose-6-phosphate isomerase), Rv0363c (fructose-bisphosphatealdolase), and Rv3255c (mannose-6-phosphate isomerase). It was found to be interacting with Rv1449c (transketolase) and Rv1448c (transaldolase) as well. These enzymes have direct involvement in shikimate pathway since reaction catalyzed by transaldolase, yields erythrose-4-phosphate, which is a precursor of shikimate pathway (**Figure 10**).

Rv2030c was found to be uniquely associated with Rv3903c, that is again a hypothetical protein of dormancy regulon (**Figure 9**). Rv2031c is known to act as a chaperone and our results support this phenomenon of its association with Rv0350 (dnaK; **Figure 10**), which has a role in protein folding. Moreover, it was found to be interacting with Rv2623 (universal stress protein) that functions in response to stress and is responsible for establishment of persistent latency in *Mtb* (Drumm et al., 2009). Interestingly, we observed Rv2031 to be interacting with Rv3875 (ESAT-6), that is known to play crucial role in pathogenesis (**Figure 10**) (Guinn et al., 2004). It was also found to be associated with different proteins having roles in metal ion binding, transcription regulation, urea catabolism, nitrate assimilation, ABC transport, and oxidoreductase activity; indicating its role as a master regulator during latency (**Figure 9**). Our study revealed network interactions and signaling pathways involved during persistence of *Mtb* during latency, which might also be helpful in defining the functionality of unknown proteins.

Integrating our results and very well supported by published literature, we propose that these proteins interact among themselves and other proteins to regulate various pathways like glycolysis that is critical for persistence of *Mtb*, pentose phosphate and shikimate, in order to adapt to stress conditions (Marrero et al., 2013). The overall model is depicted in **Figure 10**.

Stress conditions such as hypoxia, high nitric oxide (NO), low Fe, low pH, etc., induces upregulation of Rv2028–Rv2031 proteins (Yuan et al., 1996; Sherman et al., 2001). Rv2031c acts as chaperone and prevents misfolding of proteins and it interacts with dnaK (Rv0350), which is again a molecular chaperone. Thus, both the proteins perform a collaborative function to maintain structural integrity of other *Mtb* proteins and preventing them from getting denatured during stress. Rv2031 also interacts with universal stress protein TB31.7 (Rv2623) that regulates *Mtb* growth during latency. Rv2028c or universal stress protein interacts with histidine kinase receptor (Rv2027c), which has a kinase activity and helps in activation of DevS–DevR two component system and subsequent expression of latency associated genes. Thus Rv2028c indirectly modulates the gene expression during latency and helps in display of genes, which are capable to combat stress. Rv2029c is endowed with phosphofructokinase activity and regulates carbohydrate and amino acid metabolism by interacting with different enzymes that catalyze glycolysis, pentose phosphate, and shikimate pathways. *Mtb* can very well adapt during adverse conditions by regulating carbohydrate and amino acid metabolism for its survival in dormant stage. Likewise, Rv2030c has an important role in nucleotide metabolism. Dormant *Mtb* infection and reactivation is related to oxygen tension where a restricted growth is observed under hypoxic conditions and vice-versa (Ortega et al., 2014). Hence, Rv2030c which is upregulated in dormancy may play an important role in regulating growth and replication of bacterium as a response to the environment created by host immune system.

### Antigenicity Index

Latency associated antigens can be ideal vaccine candidates to target *Mtb* in its dormant state. Consequently, they can be quite crucial in providing sterile immunity. Therefore, we tried to identify probable antigens among the set of proteins, using VaxiJen webserver. Query proteins showing antigenicity index above a threshold of 0.4 were considered as probable antigens. Two out of the four proteins Rv2029c and Rv2031c were already reported to induce IFN-γ production and their role as vaccine candidates has been well established (Shi et al., 2003; Leyten et al., 2006). Our results complement with the published literature and indicate that Rv2029c and Rv2031c have antigenicity indices 0.5086 and 0.8067, respectively. On the basis of antigenic index, we observed that Rv2028c with antigenicity index, 0.6368 may act as a novel vaccine candidate. To the best of our knowledge, there is no report demonstrating the role of Rv2028c in vaccination. In contrast, Rv2030c, with a score of 0.3451 was found to possess non-antigenic properties (**Table 1**).

## Discussion

*Mycobacterium tuberculosis* modulates its growth and metabolism during the latent phase of infection to survive within the hostile environment of its host. Proteins *viz.* Rv2028c, Rv2029c, Rv2030c, and Rv2031c are known to be prominently upregulated during latency; suggesting their importance in contributing to the establishment of latent state of the bacterium (Sherman et al., 2001). Therefore, structural and functional analysis along with interactome study is of utmost importance to understand the network of biological pathways regulated by these proteins. In the present study, *in silico* analysis combined with a literature survey revealed probable functional roles and a synergistic pathway regulated by Rv2028–Rv2031 proteins. The proposed pathway provides a platform for further experimental research to understand latent TB infection. In addition, the predicted theoretical structures of the proteins may act as filler for the non-existence of experimental structures and will aid in structural biology research.

Our study predicts the evolutionary pattern of Rv2028–Rv2031 proteins among the selected mycobacterial species and probable pathways regulated during latency, based on functional annotation and protein–protein interactions. Phylogenetic analysis reveals co-evolution of query proteins in *Mtb and M. bovis,* which are strict pathogens and slow growing bacteria, indicating that these proteins may regulate the same biochemical pathways in both the mycobacterial species. Besides, phylogeny results revealed significant differences in the proteins sequences of *M. gilvum* that is a non-pathogenic, rapidly growing bacteria and *M. intracellulare,* which is an opportunistic pathogen and has a slow growth rate. Therefore, it would also provide interesting information regarding the role of proteins in the pathogenicity and growth of mycobacterial species.

The current study reveals that Rv2028–Rv2031 is important in response to stress and dormant state of *Mtb*, proteins encoded by this operon may lead to expression of latency associated genes, nucleoside metabolism, and protein folding. Rv2029c may play an important role in persistent infection by facilitating glycolysis which is crucial for persistence of *Mtb* (Marrero et al., 2013). Given the fact that these proteins are upregulated during latency, it is conceivable that they are important for the bacterium to adjust its growth, metabolism, and replication process for survival in hostile conditions. Our results lay a foundation for further research to be carried out with respect to these proteins and in understanding regulatory factors involved in the latent survival of *Mtb,* which is not yet fully studied.

The network analysis performed in this study overlaps with the protein–protein interaction data that can be accessed using TB database. Still, our results have advantage over the already existing data due to following considerations.

(i) Statistical analysis to estimate the importance of a given protein or protein–protein interaction in the network over others e.g., Rv2031c which has a high betweenness centrality value is represented by a larger node size in **Figure 9**. Likewise, protein–protein interactions of more importance are deduced on the basis of edge betweenness values.
(ii) Manual selection of high-confidence predictions, while ignoring those with low confidence.
(iii) Integrated network analysis of all query proteins at the same time provides an overall picture of protein–protein interaction between the same operonic proteins, unlike TB database that predicts interactions for a single protein.

In context of the aforementioned points, we conclude that the predictions in our analysis are of high quality and provide a better picture of protein network analysis than TB database.

In essence, our study indicates the importance of Rv2028–Rv2031 proteins in imparting flexibility to *Mtb* to survive in the intimidating surroundings of the host. The results, presented in this study are based on *in silico* prediction, we therefore, have carefully selected computational methods based on their performance and accuracy. A similar approach has already been used for characterization of Type VII secretion system in *Mtb* (Das et al., 2011). Moreover, our study points out the involvement of glycolysis in latency is consistent with the previous published study where phosphorylation of glucose, the very first step of glycolysis is indispensable for *Mtb* persistence within mouse (Marrero et al., 2013). Likewise, our study is further strengthened by yet another published study where the depletion of fructose bisphosphate aldolase (Rv0363c, one of the key component of our proposed interacting network operating during latency) resulted in the failure of *Mtb* to persist within the host (Puckett et al., 2014). Hence, these results can be relied for further experimental validation. Further, we have also predicted the antigenic potential of query proteins and our results suggest that Rv2028c can be an ideal vaccine candidate to eliminate *Mtb* in latency. Furthermore, these proteins can serve as potential drug targets owing to their biological importance.

## Conflict of Interest Statement

The authors declare that the research was conducted in the absence of any commercial or financial relationships that could be construed as a potential conflict of interest.

## Footnotes

1 http://www.uniprot.org

2 http://blast.ncbi.nlm.nih.gov/Blast.cgi

3 https://www.ebi.ac.uk/Tools/msa/clustalo

4 http://www.ebi.ac.uk/GOA

5 http://revigo.irb.hr/

6 http://www.ncbi.nlm.nih.gov/Structure/cdd/cdd.shtml

7 http://prodom.prabi.fr/prodom/current/html/home.php

8 http://www.ebi.ac.uk/interpro

9 http://psort.hgc.jp

10 http://www.cbs.dtu.dk/services/TMHMM

11 http://www.ch.embnet.org/software/TMPRED_form.html

12 http://www.cbs.dtu.dk/services/SignalP

13 http://zhanglab.ccmb.med.umich.edu/I-TASSER

14 http://string-db.org

15 http://www.ddg-pharmfac.net/vaxijen/VaxiJen/VaxiJen.html
