# Supplementary material for: Rv2031c of Mycobacterium tuberculosis: a master regulator of Rv2028–Rv2031 (HspX) operon
Source: Front Microbiol. 2015 Apr 27;6:351. doi: 10.3389/fmicb.2015.00351 (PMC4410610; doi:10.3389/fmicb.2015.00351)
Supplement: Supplementary file 1 [file Presentation_1.PDF]

Supplementary Material

Figure S1

A)

	M.tb	M.bovis	M.gilvum	M.vanbaalenii	M.vaccae	M.smegmatis	M.ulcerans	M.marinum	M.avium	M.avium paratuberculosis	M.intracellulare	M.indicus.pranii	M.leprae
M.tb	100	100	45.05	44.07	43.75	39.26	37.16	37.55	36.11	36.11	23.23	24.77	15.02
M.bovis	100	100	45.05	44.07	43.75	39.26	37.16	37.55	36.11	36.11	23.23	24.77	15.02
M.gilvum	45.05	45.05	100	73.78	69.12	39.1	38.43	38.43	40.49	40.49	24.73	27.18	12.72
M.vanbaalenii	44.07	44.07	73.78	100	70.68	37.64	36.08	36.08	38.06	38.06	22.11	24.76	14.91
M.vaccae	43.75	43.75	69.12	70.68	100	39.1	36.08	36.08	38.46	38.46	25.81	28.16	14.1
M.smegmatis	39.26	39.26	39.1	37.64	39.1	100	33.2	33.2	31.97	31.97	24.49	27.78	15.18
M.ulcerans	37.16	37.16	38.43	36.08	36.08	33.2	100	98.86	60.4	60.4	48.04	49.11	14.47
M.marinum	37.55	37.55	38.43	36.08	36.08	33.2	98.86	100	60.4	60.4	48.04	49.11	14.47
M.avium	36.11	36.11	40.49	38.06	38.46	31.97	60.4	60.4	100	100	53.19	55.77	13.6
M.avium paratuberculosis	36.11	36.11	40.49	38.06	38.46	31.97	60.4	60.4	100	100	53.19	55.77	13.6
M.intracellulare	23.23	23.23	24.73	22.11	25.81	24.49	48.04	48.04	53.19	53.19	100	99.02	13.1
M.indicus.pranii	24.77	24.77	27.18	24.76	28.16	27.78	49.11	49.11	55.77	55.77	99.02	100	12.77
M.leprae	15.02	15.02	12.72	14.91	14.1	15.18	14.47	14.47	13.6	13.6	13.1	12.77	100

B)

	M.tb	*M.bovis*	*M.gilvum*	*M.vanbaalenii*	*M.vaccae*	*M.smegmatis*	*M.ulcerans*	*M.marinum*	*M.avium*	*M.avium paratuberculosis*	*M.intracellulare*	*M.indicus.pranii*	*M.leprae*
M.tb	100	16.13	23.5	19.9	16.07	17.59	19.16	23.36	21.96	22.43	23.36	23.36	21.96
M.bovis	16.13	100	22.45	14.29	20.17	17.24	16.13	14.35	21.15	19.23	21.15	21.15	17.79
M.gilvum	23.5	22.45	100	18.49	20.49	18.64	18.01	17.23	16.8	18.04	18.98	18.98	18.6
M.vanbaalenii	19.9	14.29	18.49	100	11.03	13.48	21.89	25.57	25.57	24.05	21.76	21.76	24.05
M.vaccae	16.07	20.17	20.49	11.03	100	74.38	14.89	19.29	19.29	19.29	24.29	24.29	20
M.smegmatis	17.59	17.24	18.64	13.48	74.38	100	14.6	22.79	22.06	20.59	26.47	26.47	21.32
M.ulcerans	19.16	16.13	18.01	21.89	14.89	14.6	100	29.26	29.08	27.83	28.06	28.06	28.34
M.marinum	23.36	14.35	17.23	25.57	19.29	22.79	29.26	100	57.46	61.25	60.49	60.49	62.23
M.avium	21.96	21.15	16.8	25.57	19.29	22.06	29.08	57.46	100	61.59	59.68	59.68	60
M.avium paratuberculosis	22.43	19.23	18.04	24.05	19.29	20.59	27.83	61.25	61.59	100	65.31	65.31	65.3
M.intracellulare	23.36	21.15	18.98	21.76	24.29	26.47	28.06	60.49	59.68	65.31	100	100	73.07
M.indicus.pranii	23.36	21.15	18.98	21.76	24.29	26.47	28.06	60.49	59.68	65.31	100	100	73.07
M.leprae	21.96	17.79	18.6	24.05	20	21.32	28.34	62.23	60	65.3	73.07	73.07	100

C)

	M.tb	M.bovis	M.gilvum	M.vanbaalenii	M.vaccae	M.smegmatis	M.ulcerans	M.marinum	M.avium	M.avium paratuberculosis	M.intracellulare	M.indicus.pranii	M.leprae
M.tb	100	86.03	26.43	26.14	24.84	28.1	25.81	25.81	27.52	28.26	25.16	28.19	27.59
M.bovis	86.03	100	24.29	25.49	24.84	27.45	25.16	25.16	28.19	28.26	25.16	26.85	25.52
M.gilvum	26.43	24.29	100	32.85	33.09	32.93	33.17	33.17	46.23	48.11	48.11	47.66	49.08
M.vanbaalenii	26.14	25.49	32.85	100	85.9	77.35	75.99	73.84	59.38	62.44	59.56	56.44	53.81
M.vaccae	24.84	24.84	33.09	85.9	100	76.76	74.96	72.85	59.38	63.85	60.89	56.89	56.5
M.smegmatis	28.1	27.45	32.93	77.35	76.76	100	82.03	79.97	59.38	62.44	60	59.11	54.26
M.ulcerans	25.81	25.16	33.17	75.99	74.96	82.03	100	97.03	61.61	63.85	61.23	58.22	56.05
M.marinum	25.81	25.16	33.17	73.84	72.85	79.97	97.03	100	61.61	63.85	61.23	58.22	56.05
M.avium	27.52	28.19	46.23	59.38	59.38	59.38	61.61	61.61	100	84.91	85.71	66.36	66.05
M.avium paratuberculosis	28.26	28.26	48.11	62.44	63.85	62.44	63.85	63.85	84.91	100	99.54	67.3	66.51
M.intracellulare	25.16	25.16	48.11	59.56	60.89	60	61.23	61.23	85.71	99.54	100	64.71	64.81
M.indicus.pranii	28.19	26.85	47.66	56.44	56.89	59.11	58.22	58.22	66.36	67.3	64.71	100	70.64
M.leprae	27.59	25.52	49.08	53.81	56.5	54.26	56.05	56.05	66.05	66.51	64.81	70.64	100

D)

	M.tb	M.bovis	M.gilvum	M.vanbaalenii	M.vaccae	M.smegmatis	M.ulcerans	M.marinum	M.avium	M.avium paratuberculosis	M.intracellulare	M.indicus.pranii	M.leprae
M.tb	100	36.36	24.81	21.71	21.71	21.71	21.71	21.88	25.78	25.78	25.78	24.03	25.2
M.bovis	36.36	100	28.57	25.56	25.56	27.82	27.82	35.61	35.11	35.11	31.3	31.06	30
M.gilvum	24.81	28.57	100	76.03	76.03	80.14	80.14	35.25	28.57	28.57	30.71	29.08	30
M.vanbaalenii	21.71	25.56	76.03	100	100	87.67	87.67	34.06	31.43	31.43	31.43	28.57	29.5
M.vaccae	21.71	25.56	76.03	100	100	87.67	87.67	34.06	31.43	31.43	31.43	28.57	29.5
M.smegmatis	21.71	27.82	80.14	87.67	87.67	100	100	35.51	30.71	30.71	32.14	30	30.94
M.ulcerans	21.71	27.82	80.14	87.67	87.67	100	100	35.51	30.71	30.71	32.14	30	30.94
M.marinum	21.88	35.61	35.25	34.06	34.06	35.51	35.51	100	59.57	59.57	56.03	60.14	58.16
M.avium	25.78	35.11	28.57	31.43	31.43	30.71	30.71	59.57	100	100	71.33	60.84	58.45
M.avium paratuberculosis	25.78	35.11	28.57	31.43	31.43	30.71	30.71	59.57	100	100	71.33	60.84	58.45
M.intracellulare	25.78	31.3	30.71	31.43	31.43	32.14	32.14	56.03	71.33	71.33	100	62.24	62.68
M.indicus.pranii	24.03	31.06	29.08	28.57	28.57	30	30	60.14	60.84	60.84	62.24	100	65.73
M.leprae	25.2	30	30	29.5	29.5	30.94	30.94	58.16	58.45	58.45	62.68	65.73	100

Figure S2

A)

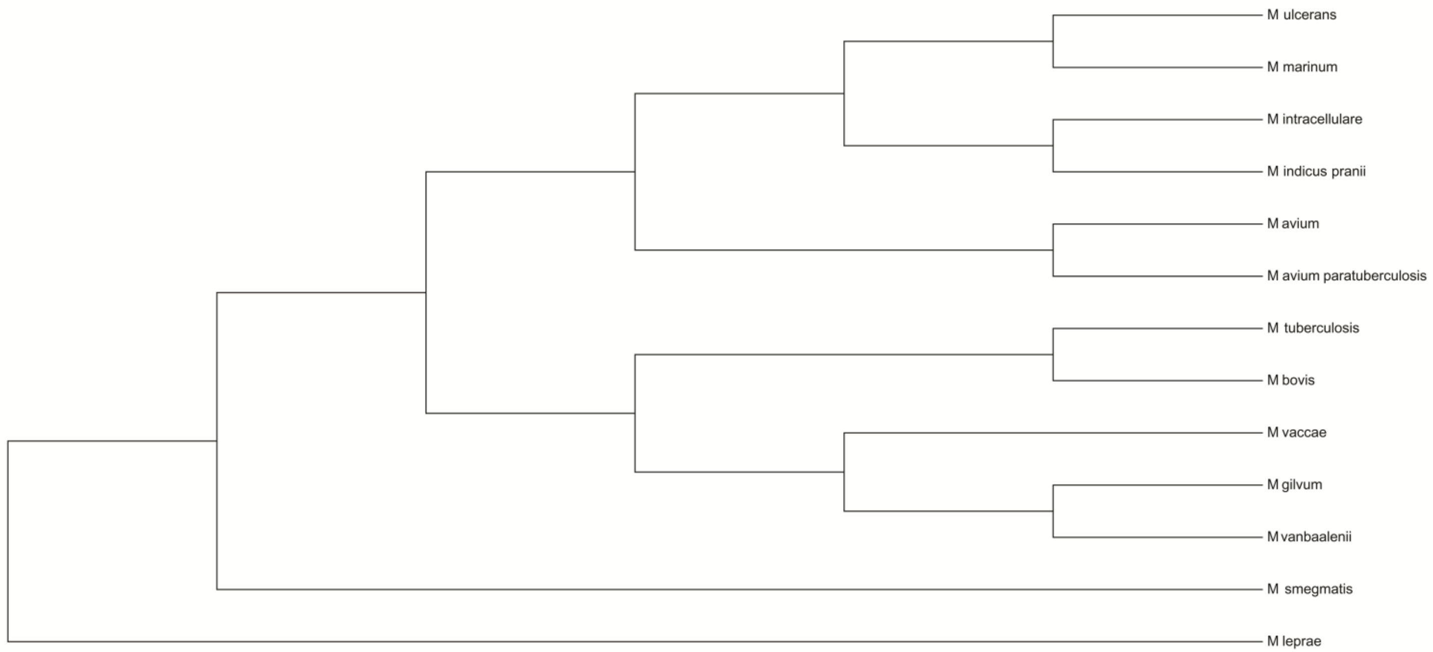
M ulcerans
M marinum
M intracellulare
M indicus pranii
M avium
M avium paratuberculosis
M tuberculosis
M bovis
M vaccae
M gilvum
M vanbaalenii
M smegmatis
M leprae

B)

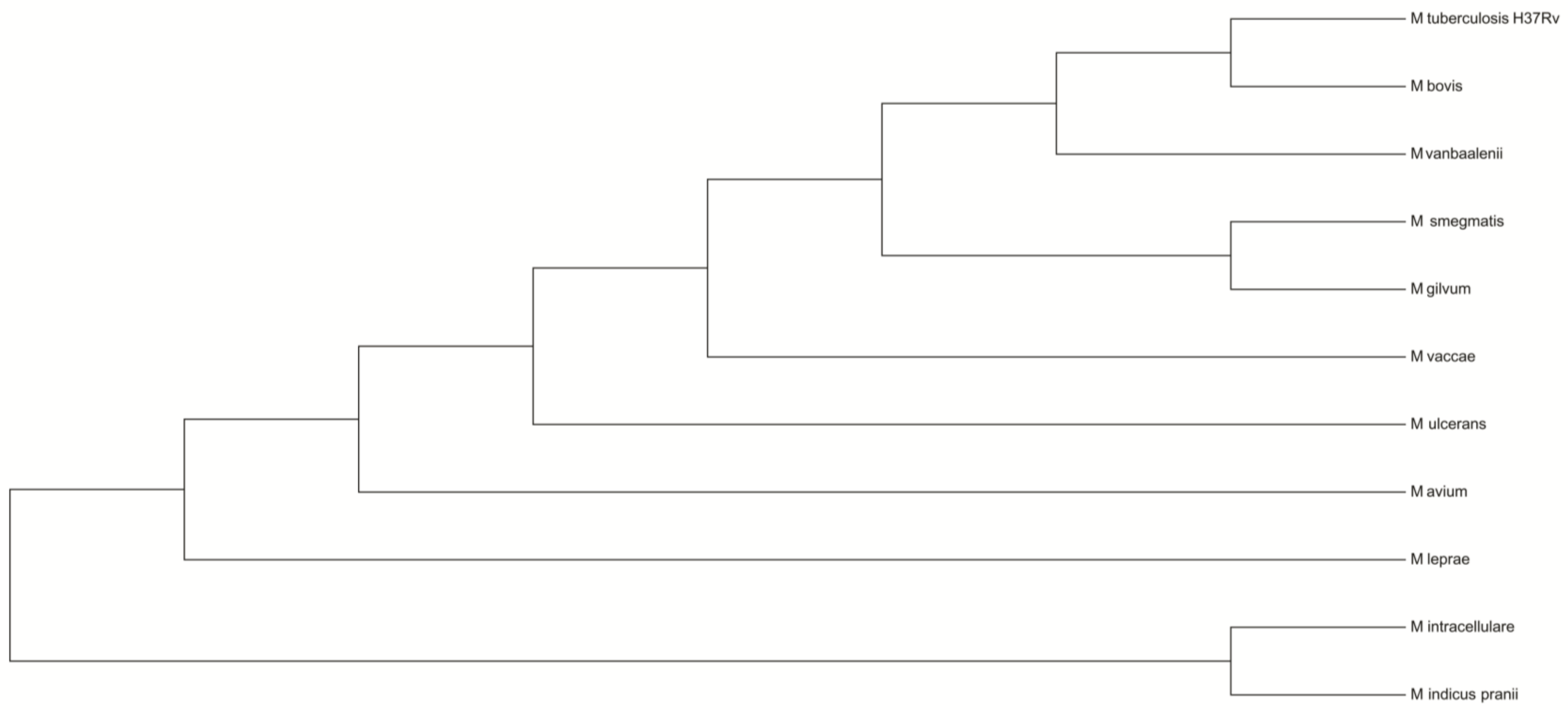

M tuberculosis H37Rv
M bovis
M vanbaalenii
M smegmatis
M gilvum
M vaccae
M ulcerans
M avium
M leprae
M intracellulare
M indicus pranii

C)

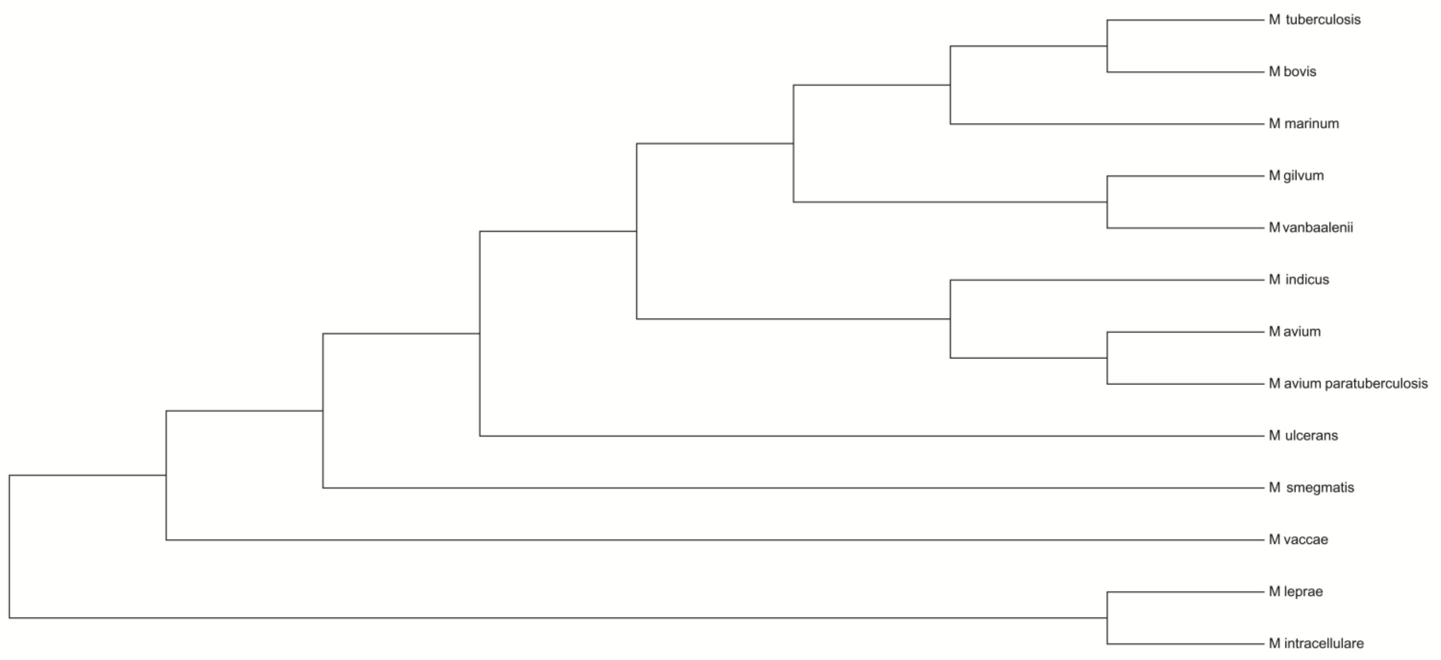
M tuberculosis
M bovis
M marinum
M gilvum
M vanbaalenii
M indicus
M avium
M avium paratuberculosis
M ulcerans
M smegmatis
M vaccae
M leprae
M intracellulare

D)

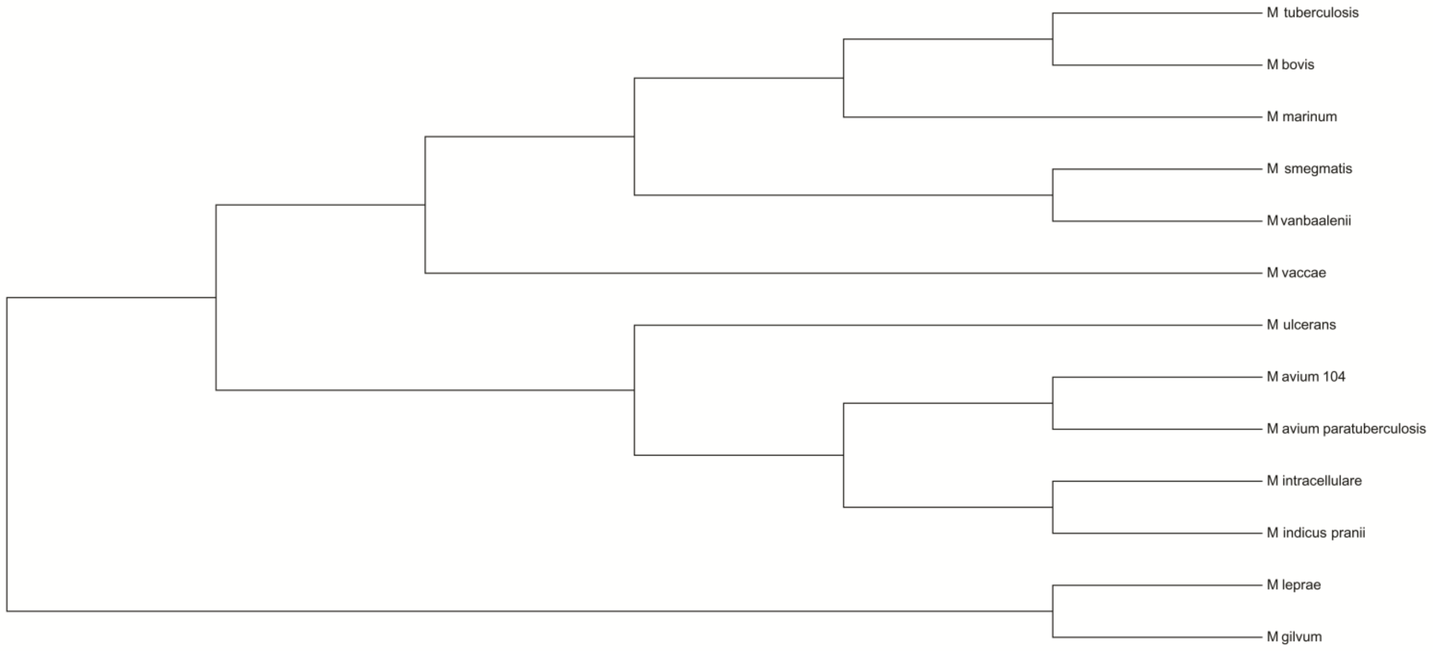
M tuberculosis
M bovis
M marinum
M smegmatis
M vanbaalenii
M vaccae
M ulcerans
M avium 104
M avium paratuberculosis
M intracellulare
M indicus pranii
M leprae
M gilvum

Figure S3

A) Universal stress family protein (RV2028c)

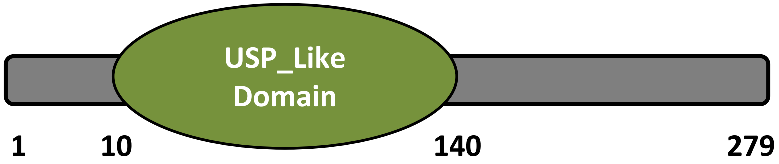

B) 6-phosphofructokinase PfkB (RV2029c)

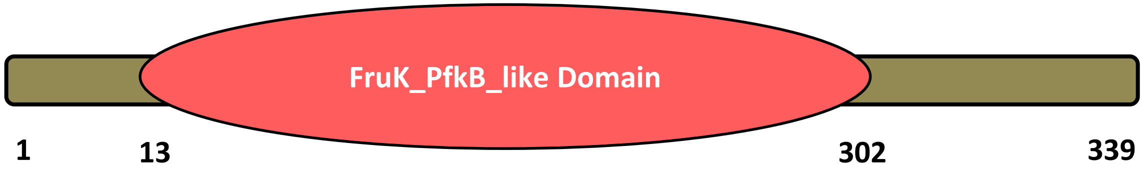

C) Conserved protein (RV2030c)

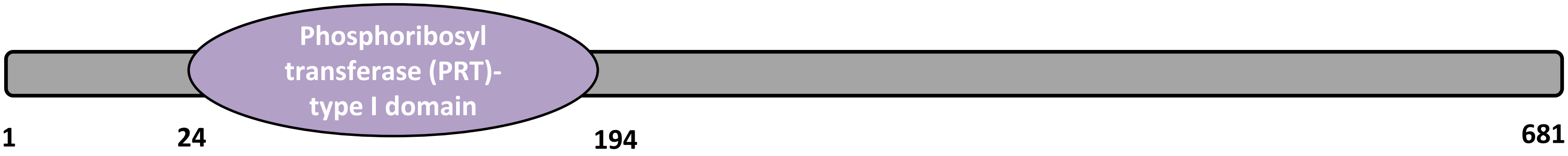

D) Heat shock protein HspX (RV2031c)

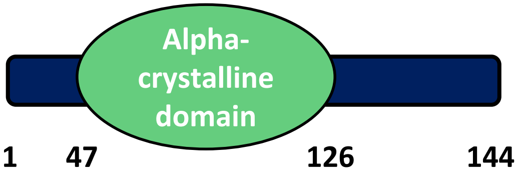

Table S1

**Mycobacterium Species**	**Pathogenicity**	**Growth Rate**
Mycobacterium tuberculosis	Strict pathogen	Slow
Mycobacterium bovis	Strict pathogen	Slow
Mycobacterium leprae	Strict pathogen	Slow
Mycobacterium ulcerans	Strict pathogen	Slow
Mycobacterium marinum	Strict pathogen	Slow
Mycobacterium avium	Opportunistic pathogen	Slow
Mycobacterium intracellulare	Opportunistic pathogen	Slow
Mycobacterium avium subsp. paratuberculosis K-10	Opportunistic pathogen	Slow
Mycobacterium indicus pranii	Non pathogen	Slow
Mycobacterium smegmatis	Non pathogen	Rapid
Mycobacterium gilvum	Non pathogen	Rapid
Mycobacterium vanbaalenii	Non pathogen	Rapid
Mycobacterium vaccae	Non pathogen	Rapid

Legends

Figure S1: *Percent-identity matrix for proteins encoded by Rv2028c-Rv2031c operon showing sequence identity to proteins in different mycobacterial species.* Figure represents percent identity matrix for A) Rv2028c; B) Rv2029c; C) Rv2030c; D) Rv2031c. Source organism for different proteins are written in horizontal and vertical axes of the matrix with each cell containing percent identity values between corresponding proteins. Red, yellow and green cell colour represents percent-identity values below 20%, 20-35% and above 35%, respectively.

Figure S2: *Consensus phylogenetic tree of proteins encoded by Rv2028c-Rv2031c operon after bootstrapping.* Figure represents phylogenetic trees for A) Rv2028c; B) Rv2029c; C) Rv2030c; D) Rv2031c constructed using neighbour joining method. Mycobacterial species sharing common ancestry are placed in the sister groups forming different taxon.

Figure S3: *Predicted functional domains in proteins expressed by Rv2028c-Rv2031c operon genes.* A) Universal stress family protein like domain is found in proteins whose expression is enhanced during stress conditions. These proteins are important for survival during prolonged exposure to such conditions; B) phosphofructokinase and other sugar kinases like domains play an important role in carbohydrate metabolism; C) phosphoribosyl transferase (PRT)-type I domain is related to nucleotide synthesis; D) alpha-crystalline domain is found in stress induced proteins, which act as ATP-independent chaperon. The corresponding name of the proteins and sequence length is displayed as a pictorial representation.

Table S1: *Mycobacterial species selected for the study.* Table summarizes the selected mycobacterial species, along with their level of pathogenicity and growth rate.
